# Female Breast Cancer Patients, Mastectomy-Related Quality of Life: Experience from Ethiopia

**DOI:** 10.1155/2020/8460374

**Published:** 2020-04-09

**Authors:** Engida Abebe, Kassaw Demilie, Befekadu Lemmu, Kirubel Abebe

**Affiliations:** Department of Surgery, SPHMMC, Addis Ababa, Ethiopia

## Abstract

**Background:**

Mastectomy is the most common form of treatment for a developing-nation woman diagnosed with breast cancer. This can have huge effect on a women's quality of life.

**Objective:**

To assess mastectomy-related quality of life in female breast cancer patients.

**Materials and Methods:**

A facility-based cross-sectional descriptive study was conducted from February 1^st^ to July 30^th^, 2018. A pretested structured data collection format was used to interview patients. The European Organization for Research and Treatment for Cancer Quality of Life Questionnaire-Core 30 (EORTC QLQ-C30) and Breast Cancer-Specific (EORTC QLQ-BR23) were used to evaluate quality of life, functional capacity, and symptom scales. Data was analyzed with SPSS version 23.

**Results:**

The mean age of the 86 patients was 43.2 years (SD ± 11.4) and ranged from 25 to 70 years. 54.7% (47) of patient's mastectomy was done on the right side. Based on EORTC QLQ-C30 global health status/QOL scale, the mean score was 48.3. On the evaluation of EORTC QLQ-BR23, future perspective about their health was low with a mean of 40.3 and their sexual functioning and enjoyment were significantly affected with mean scores of 85.3 and 71.2, respectively. Symptom scales were low with mean from 19.1 to 24.5. Majority (49, 57%) of respondents do not want to have breast reconstruction after mastectomy.

**Conclusion:**

Our breast cancer patients who underwent mastectomy performed poor in terms of quality of life as compared to international findings which demands attention in incorporating psychosocial aspects in the treatment plan.

## 1. Background

Breast cancer (BC) is a potentially deadly disease affecting one in eight women. It is the most frequent cause of cancer death in less-developed regions, causing one in five deaths in African women and 50–75% of women present with very advanced disease [1, 2]. According to World Health Organization Cancer Country Profile 2014, incidence of breast cancer in Ethiopia was reported to be 12,956, contributing to 24.4% of the deaths [3]. Even if adequate data is lacking in the trends of BC in Ethiopia, some authors suggested that it is increasing [4].

Surgery is the primary modality in the management of resectable BC. In certain parts of the world including Africa, mastectomy can be the only treatment option due to limited resources for complimentary adjuvant therapies [5]. Reports from east Africa indicate that up to 99% of patients undergo mastectomy for a lack of other modalities of treatment [6].

As Ethiopia is a developing nation, adjuvant treatment for BC do exists but not readily available. There is only one radiotherapy machine for a population of 110 million which makes adjuvant, neoadjuvant, or breast conserving surgery inaccessible. Due to the above-mentioned reasons, the main stay of treatment modality for BC in Ethiopia is modified radical mastectomy. Despite the current effort of the Ethiopian government on the issue of noncommunicable diseases including cancer, there is no screening program for BC in the country [7].

In general, survival of women with BC in Sub-Saharan Africa tends to be poor due to a number of reasons such as late presentation and poor access to timely and standard treatment [8]. According to a retrospective follow-up study with survival analysis done by Areri et al. at a teaching hospital, Adult Oncology Unit, Addis Ababa, Ethiopia, it showed that the overall estimated survival rate after diagnosis of BC was 26.42% at 72 months of follow-up [9].

As breast is considered as an attribute of feminity, maternity, and sexuality, its loss as a remedy for breast cancer can affect quality of life of women. When evaluating holistically the life of woman after mastectomy, all spheres of everyday functioning should be taken into account including physical, cognitive, emotional, and social wellbeing [10].

Traditionally, the primary end points in evaluations of medical therapies were improvement in clinical outcomes, cure, and survival. However, the concept of the medical outcome's movement and the worldwide effort to contain the rising costs of care has underscored the importance of patient-centered outcomes. There are different parameters which were used to assess quality of life (QOL) of patients with chronic illness including health-related quality of life (HRQOL). HRQOL is one of several variables commonly studied in the field of medical outcomes research. It encompasses a wide range of human experience, including functioning and subjective responses to illness. HRQOL instruments may be general or disease-specific. General HRQOL domains address the components of overall wellbeing, whereas disease-specific domains focus on the impact of particular organic dysfunctions that affect HRQOL [11]. Examples of cancer-specific instruments include the European Organization for the Research and Treatment of Cancer Quality of Life Questionnaire (EORTC QLQ-C30) [12].

Even though significant numbers of mastectomies are done in Ethiopia, to the best of the authors' knowledge, there are no data which assessed the quality of life of patients after mastectomy at a national level. Hence, this study was conducted to assess the quality of life in female breast cancer patients who underwent mastectomy.

## 2. Materials and Methods

A cross-sectional study was conducted from February 1^st^ to July 30^th^, 2018, at St. Paul's Hospital Millennium Medical College (SPHMMC), a teaching-tertiary referral hospital in Addis Ababa. Patients are followed postoperatively by consultant surgeons, oncologists, and surgery residents. All female breast cancer patients who underwent mastectomy at SPHMMC were included while male breast cancer patients, female patients operated elsewhere, and patients who had mastectomy for nonmalignant conditions were excluded.

Data was collected by trained OPD nurses using a pretested questionnaire. Data on sociodemographic characteristics (age, marital status, parity, ethnicity, educational background, and residency) and side of mastectomy were collected in as shown in Background. In addition, selected items of EORTC QLQ-C30 and EORTC QLQ-BR23 were adopted and used to asses HRQOL, psychosocial and functional scales, and postmastectomy symptoms. During data collection, the principal investigator checked data for completeness, any ambiguity, and suspicions on the spot. Collected data was cleaned, checked for completeness, entered to EpiData 3.1, and exported and analyzed with SPSS version 23. Mean scores of EORTC QLQ-C30 and EORTC QLQ-BR23 were calculated. Then QOL, functional scales, and symptom scales were calculated. Ethical clearance was obtained from SPHMMC IRB. Individual patient written consent to participate in the study was obtained. Confidentiality was kept throughout the study.

NB: The QLQ-C30 is composed of both multi-item scales and single-item measures. These include five functional scales, three symptom scales, a global health status/QOL scale, and six single items. All of the scales and single-item measures range in score from 0 to 100. Range is the difference between the maximum possible value of raw scores (RS) and the minimum possible value. The QLQ-C30 has been designed so that all items in any scale take the same range of values. Therefore, the range of RS equals the range of the item values. Most items are scored 1 to 4, giving range = 3. The exceptions are the items contributing to the global health status/QOL, which are 7-point questions with range = 6. A high scale score represents a higher response level. Thus, a high score for a functional scale represents a high/healthy level of functioning except in sexual functioning and enjoyment. High score for the global health status/QOL represents a high QOL, but a high score for a symptom scale/item represents a high level of symptomatology/problems.

## 3. Results

### 3.1. Sociodemographic Characteristics

**Figure 1:**
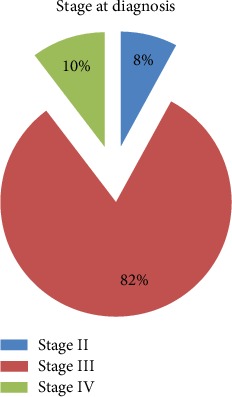
Stages of breast cancer at diagnosis (SPHMMC, 2018).

**Figure 2:**
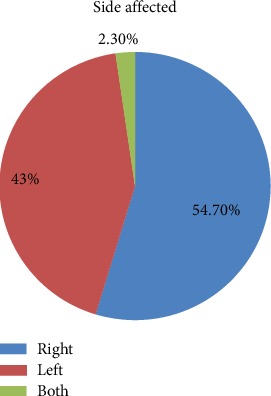
Site of affected breast (mastectomy done) (SPHMMC, 2018).

A total of 86 patients with breast cancer were included. The mean and median age of patients was 43.23 years (SD ± 11.35) and 42 years and ranged from 25 to 70 years. The majority of the patients were Amhara (34, 39.5%) and Oromo (31, 36%) in ethnicity. Nearly one-third of the patients (28, 32.6%) did not attend any formal education while 20 (23.3%) attended college/university. More than half (44, 51.2%) of the patients were housewives. Forty-seven (54.7%) participants are married and 71 (82.6%) had children, with majority (58, 81.7%) having two or more children. Urban residents accounted for 73.3% (63) of the patients (Table 1). Majority (70, 81.8%) of them were diagnosed with stage III disease, and all of them have mastectomy. More than half (47, 54.7%) of the patients had the cancer on the right side (Figures 1 and 2).

### 3.2. Quality of Life Assessment

Based on EORTC QLQ-C30, the global health status/QOL scale of study participants had mean and median scores of 48.25 and 48.1, respectively. When it comes to breast cancer QOL assessment (based on EORTC QLQ-BR23), the mean and median scores for body image were 69.3 and 74.6. The mean score of future perspective about their health was 40.3 and the median was 42. The mean and median scores for sexual functioning were 85.3 and 89.6, while that of sexual enjoyment were 71.2 and 73.2, respectively.

Postoperative breast symptoms scale had mean and median scores of 19.1 and 15.3 while arm symptoms had mean and median scores of 24.5 and 20.3, respectively (Table 2).

Patients who are >50 years (*p* = 0.044), unemployed (*p* = 0.013), no formal education (*p* = 0.044), married (*p* = 0.005), and living in urban area (*p* = 0.013) had significantly higher body image response level than their respective groups. Regarding GHS/QOL and symptom scores, there is a significant response level difference among the different demographic variables (Table 3).

Majority (49, 57%) of the respondents do not want to have breast reconstruction after mastectomy.

## 4. Discussions

In agreement with other studies, breast cancer affected younger patients in urban setting [2, 5, 6]. And the right breast was more commonly involved than the left. Though it is a cross-sectional study which assessed patients' QOL at one point, overall, patients' quality of life was low compared to that in literatures. For example, the mean score for QOL according to EORTC QLQ-C30 for the study patients was 48.25, which is low compared to a study in Poland which showed a mean score of 68.33 and 84.23, one month and one year after mastectomy, respectively [13].

It is also lower compared to the finding of Costa et al. in Brazil who analyzed GHS patients for different stages. The mean score of GHS of patients without metastasis was 62 (SD = 24) points, while those with locoregional metastases was 63 (SD = 21.4), and the distant metastasis was 51.3 (SD = 24) points [14]. This finding makes the mean scores of our patients' (all stages combined) QOL even worse than those of patients with advanced breast cancers.

The lower mean score of our patients may be related partly to the study design which assessed the QOL observed at single point. But it is likely to be due to the lack/absence of formal psychological and social support by a trained personnel. The economic impact of the cancer care in a setting where treatment is out of pocket can also be huge in patient's postoperative psychosocial performance. The strong family attachments among Ethiopians can be a good opportunity to train families on how to support cancer patients socially and psychologically.

On assessment of the scales/items of EORTC QLQ-BR23 functional scale, our patients had higher mean score of body image compared to studies in Turkey, Sudan, and England. The Turkey study showed that mastectomy negatively affected a woman's body image and her self-image [15]. A study conducted in Khartoum showed that patients after mastectomy were unsatisfied with their body images initially but improved over time [16]. Similarly, in England, they found out that body dissatisfaction has become an issue for women with breast cancer, who usually undergo several treatments which alter their appearance. These body image concerns can have a profound impact on quality of life, which can persist for years following recovery [17]. The higher mean score of body image in our patients can partly be attributed to different coping mechanisms practiced like by letting feelings out somehow, having religious attachments, and accepting as if nothing could be done. In our study, majority were married and housewives; they may isolate themselves from the public to decrease the psychological burden about their body image [18].

Our patients' future perspective mean score was lower compared to an Indian study which showed a future perspective mean score of 72.62 [19]. This means they are worried about their future health even though they underwent mastectomy with a cure intent. This might be related to the stage at presentation which is higher and the capacity of the health facilities which are inadequate and difficult to access.

The high mean score in sexual desire, satisfaction, and enjoyment as assessed by EORTC QLQ-BR23 means that the practice of sexual intercourse and satisfaction was affected negatively. This score was higher compared to a study conducted in Khartoum. The Khartoum study showed sexual pleasure to be not affected by mastectomy, and most of the patients became more active and satisfied sexually over time [16]. This can be explained by most of our study participants who are in their prime age of reproduction, are married, did not attend formal education, have limited communication and social interaction with health care professionals, and have a lack of psychological support. The mean score of postoperative breast and arm symptoms were low, which means our patients were not suffering from complications related to mastectomy site and ipsilateral arm. This finding is comparable to a study found in Poland but higher than a study found in India which showed mean scores of 8.98 and 15.52 for breast and arm symptoms [13, 18].

Overall, our breast cancer patients who underwent mastectomy performed poor in terms of quality of life as compared to international findings which demands attention in incorporating psychosocial aspects in the treatment plan. In addition, this probably could have been improved if patients presented early and breast conserving surgery was available. Designing and implementing screening programs at all levels can help improve overall outcome and quality of life of patients who underwent modified radical mastectomy. Adjuvant treatment including radiotherapy availability at least at referral hospitals can make a difference in the way breast cancer patients are managed and can improve their quality of life after the care. As this study has a relatively small subject studied in a cross-section, the true picture needs to be assessed with a further study which assesses quality of life of cancer patients in general and breast cancer patients in particular which includes multiple centers including a bigger study subject.

## Figures and Tables

**Table 1:** Sociodemographic characteristics of female breast cancer patients, mastectomy-related quality of life (SPHMMC, Addis Ababa, Ethiopia, 2018).

Item	Age range	Number	Percent
Age distribution	≤30	17	19.8%
	31-40	24	27.9%
	41-50	24	27.9%
	51-60	14	16.3%
	>60	7	8.1%

Ethnicity	Amhara	34	39.5%
	Oromo	31	36%
	Tigre	6	7.0%
	Gurage	5	5.8%
	Others	10	11.6%

Occupation	Housewife	44	51.2%
	Government employee	15	15.1%
	Merchant	9	10.5%
	Others	20	23.3%

Marital status	Married	47	54.7%
	Single	9	10.5%
	Divorced	18	14.0%
	Widowed	12	20.9%

Educational status	Illiterate	28	32.6%
	Read and write	10	11.6%
	Elementary school	10	11.6%
	High school	18	20.9%
	College/university	20	23.3%

Residency	Urban	63	73.3%
	Rural	23	26.7%

Parity	Yes	71	82.6%
	No	15	17.4%

**Table 2:** QOL, functional capacity, and symptom scales of female breast cancer patients, mastectomy-related quality of life (SPHMMC, Addis Ababa, Ethiopia, 2018).

Scale	Scale	Mean	Median
EORTC QLQ-C30^1^	GHS/QOL^2^	48.25	48.1
EORTC QLQ-BR23^3^	Functional scales		
	Body image	69.3	74.6
	Future perspective	40.3	42
	Sexual functioning^∗^	85.3	89.6
	Sexual enjoyment^∗^	71.2	73.2
	Symptom scales/items		
	Breast symptoms	19.1	15.3
	Arm symptoms	24.5	20.3

^1^European Organization for the Research and Treatment of Cancer Quality of Life Questionnaire. ^2^GHS/QOL: global health status/quality of life. ^3^EORTC QLQ-BR23: European Organization for Research and Treatment Center Quality of Life Questionnaire Breast Cancer-Specific. NB: Items for the scales marked ^∗^ are scored positively (i.e., “very much” is best) and therefore use the same algebraic equation as for symptom scales; however, the body image scale uses the algebraic equation for functioning scales.

**Table 3:** Association between demographic characteristics and QOL, functional capacity, and symptom scales of female breast cancer patients, mastectomy-related quality of life (SPHMMC, Addis Ababa, Ethiopia, 2018).

Variables		*n* = 86	Body image		Future perspective		Sexual functioning		Sexual enjoyment		Breast symptoms		Arm symptom		QOL	
			Scale	*p* value	Scale	*p* value	Scale	*p* value	Scale	*p* value	Scale	*p* value	Scale	*p* value	Scale	*p* value
Age	≤50	59	63.7	0.044	38.9	0.020	87.3	0.018	83.3	0.113	17.0	0.070	27.6	0.108	48.7	0.011
	>50	27	74.7		42.3		83.6		59.2		22.0		20.8		48.1	

Occupation	Employed	42	67.4	0.013	39.0	0.013	85.0	0.018	57.6	0.120	17.6	0.036	25.6	0.057	49.5	0.027
	Unemployed	44	71.2		41.6		85.6		84.8		20.6		23.2		46.6	

Educational status	No formal education	28	73.5	0.044	50.0	0.164	94.6	0.074	94.0	0.205	18.8	0.009	24.6	0.250	49.1	0.016
	Formal education	58	65.1		30.6		76.0		48.4		19.3		23.8		47.7	

Marital status	Married	77	74.3	0.005	41.1	0.210	87.0	0.017	87.9	0.153	18.7	0.005	25.0	0.280	49.0	0.014
	Single	9	64.3		39.5		83.6		54.5		19.4		24.0		48.0	

Residency	Urban	63	70.1	0.013	40.7	0.014	87.6	0.021	87.8	0.153	17.3	0.057	23.9	0.001	49.0	0.021
	Rural	23	68.5		40.0		83.0		54.6		20.9		25.0		48.0	

Parity	No parity	15	67.2	0.013	34.2	0.007	83.8	0.007	53.7	0.154	20.2	0.058	24.6	0.021	48.2	0.009
	Parity	71	71.4		46.4		86.7		88.7		18.0		24.1		47.9	

## Data Availability

The data used in the manuscript are available in their respective journals.
